# Screening for Methane Utilizing Mixed Communities with High Polyhydroxybutyrate (PHB) Production Capacity Using Different Design Approaches

**DOI:** 10.3390/polym13101579

**Published:** 2021-05-14

**Authors:** Rana Salem, Moomen Soliman, Ahmed Fergala, Gerald F. Audette, Ahmed ElDyasti

**Affiliations:** 1 Civil Engineering Department, York University, 4700 Keele Street, Toronto, ON M3J 1P3, Canada; rsalem@yorku.ca (R.S.); ilasimbf@yorku.ca (M.S.); 2 Department of Biotechnology, Delft University of Technology, Mekelweg 5, 2628 CD Delft, The Netherlands; a.m.a.a.fergala@tudelft.nl; 3 Chemistry Department, York University, 4700 Keele Street, Toronto, ON M3J 1P3, Canada; audette@yorku.ca

**Keywords:** wastewater, bioplastics, methane, polyhydroxyalkanoate (PHA), methanotrophs, polyhydroxybutyrate (PHB)

## Abstract

With the adverse environmental ramifications of the use of petroleum-based plastic outweighing the challenges facing the industrialization of bioplastics, polyhydroxyalkanoate (PHA) biopolymer has gained broad interest in recent years. Thus, an efficient approach for maximizing polyhydroxybutyrate (PHB) polymer production in methanotrophic bacteria has been developed using the methane gas produced in the anaerobic digestion process in wastewater treatment plants (WWTPS) as a carbon substrate and an electron donor. A comparison study was conducted between two experimental setups using two different recycling strategies, namely new and conventional setups. The former setup aims to recycle PHB producers into the system after the PHB accumulation phase, while the latter recycles the biomass back into the system after the exponential phase of growth or the growth phase. The goal of this study was to compare both setups in terms of PHB production and other operational parameters such as growth rate, methane uptake rate, and biomass yield using two different nitrogen sources, namely nitrate and ammonia. The newly proposed setup is aimed at stimulating PHB accumulating type II methanotroph growth whilst enabling other PHB accumulators to grow simultaneously. The success of the proposed method was confirmed as it achieved highest recorded PHB accumulation percentages for a mixed culture community in both ammonia- and nitrate-enriched media of 59.4% and 54.3%, respectively, compared to 37.8% and 9.1% for the conventional setup. Finally, the sequencing of microbial samples showed a significant increase in the abundance of type II methanotrophs along with other PHB producers, confirming the success of the newly proposed technique in screening for PHB producers and achieving higher PHB accumulation.

## 1. Introduction

Environmental protection and wellbeing have become a major concern in recent years. Currently, one of the most commonly studied environmental topics is plastic disposal and the extent to which it poses a high-risk threat due to its subsequent accumulation in the environment [1]. Petroleum-based plastics are nonbiodegradable and have long half-lives, estimated to be hundreds of years, which lead to severe ecological repercussions, such as toxicity to living organisms including humans and disruption of marine ecosystems [1,2]. Despite the successful advancement in plastic manufacturing, it still has a crucial environmental drawback of resistance to degradation. The existing routes of plastic disposal are inefficient as they mainly dispose of plastics into landfills or aquatic surfaces, which means these synthetic polymers can persist in the environment for thousands of years [3].

Thus, there has been an evolutionary approach to find a more suitable ecofriendly biodegradable polymer alternative, i.e., bio-based plastic, that can be synthesized from nature-acquired resources, such as the plant-based polylactic acid (PLA) and the bacteria-based polyhydroxyalkanoate (PHA). PHA polymers can be produced using sustainable renewable waste materials, unlike petroleum-based plastics, which are produced from nonrenewable fossil fuels [4]. PHAs are microbially synthesized hydrophobic inclusions that are stored intracellularly under stress conditions of phosphorus, nitrogen, and oxygen limitations accompanied by carbon abundance [5]. These polymers are nontoxic, biocompatible, and completely break down into water and carbon dioxide under aerobic conditions or into methane under anaerobic conditions, making them safe to dispose into the environment without any dangerous or unpredictable outcomes [6].

PHA could replace petroleum-based plastics in the near future owing to the fact that this polymer offers an alternative to overcome the aforementioned problems associated with petroleum-based plastics [7]. However, there are some limitations facing the process of PHA industrialization or upscaling, with the most common being its production cost [8]. Synthesizing PHA polymer is a cost-intensive process due to the high cost of carbon feedstock, design of culturing operational conditions, and the polymer extraction process [9]. Studies on PHA production have been performed using expensive carbon substrates such as glucose sugar, which comprises over 40% of the production cost [10]. Hence, more research should be focused on using waste renewable feedstock for PHA accumulation. Furthermore, studies have also been conducted on the development of less expensive culturing strategies and recovery approaches in an attempt to minimize costs [11,12].

Interestingly, it has been found that a certain type of methanotrophic bacteria has the ability to accumulate PHB through the oxidation of methane gas [13]. Methane is produced as a waste product in the anaerobic digestion process in wastewater treatment plants. The use of methanotrophs to mitigate the methane released would cut the production cost while averting its harmful emission into the atmosphere [14,15]. 

Methanotrophs are Gram-negative mesophilic bacteria commonly found in aquatic systems [16]. Methanotrophic bacteria use a common methane oxidation pathway catalyzed by the action of methane monooxygenase enzyme to convert methane to formate. Formate is in turn assimilated in the ribulose monophosphate pathway RuMP in nutrient-sufficient conditions by type I and X methanotrophs belonging to Gammaproteobacteria to carbon dioxide and biomass. On the other hand, type II methanotrophs belonging to Alphaproteobacteria use the serine pathway in nutrient-deficient conditions to form CO_2_ and store PHB as end products [17].

Conventionally, the selection of type II methanotrophs i.e., PHB accumulators, was established using ammonia as a selective pressure, a process also known as media-based selection [18]. Typically, ammonia (NH_4_) is used as an inhibitor to type I methanotrophs as it has a chemical structure that resembles that of methane (CH_4_), and thus it acts as a competitive substrate with methane and eventually intoxicates the cell [19]. Unlike type I methanotrophs, type II methanotrophs have the proper genes to overcome the toxicity of hydroxylamine and nitrite produced by methane co-oxidation, as shown in Figure S1, which makes ammonia an optimum candidate for type II selection due to its ability to metabolize these chemical by-products. Hence, the carbon substrate conflict no longer exists for type II methanotrophs [20]. However, this conventional setup has been proven to reach a maximum threshold accumulation of 40–51% of strains [18,21].

Thus, the main focus of this research is to apply a different selection pressure to overcome the PHB accumulation percentage threshold reported in the literature. A new experimental setup that relies on recycling of the biomass after the PHB phase is proposed as opposed to the conventional setup, where biomass recycling occurs after the growth phase, thereby allowing a broad array of microorganism to coexist with the type II methanotrophic community. The rationale behind this proposal is to allow bacterial strains that can withstand the stress conditions of nutrient depletion to be constantly recycled back into the systems, which would ensure that PHB accumulators have dominance within the culture media as opposed to recycling all the biomass after the exponential phase of growth, which only relies on ammonia for selection of type II methanotrophs. This setup could replace the conventional media-based selection method by a PHB-based selection system, given nitrogen source has been proven to be a precursor that greatly affects the accumulation percentage [22]. The argument against ammonia as a cellular intoxicator is usually outweighed by its ability to select type I methanotrophs when compared with nitrate. However, nitrate as a substrate requires less energy than ammonia because it is directly metabolized to the final product [20]. Herein, a comparison study is presented between the proposed PHB-based selection and conventional nitrogen-based selection using both nitrate and ammonia as nitrogen source.

## 2. Materials and Methods 

### 2.1. Setup and Operational Condition

All the experiments were conducted in 250 mL culture bottles with a butyl rubber cap to enable gas vacuuming and subsequent injection. In each bottle, 10 mL of seed was added to 40 mL of mineral salt medium (MSM) for culture enrichment. The bottles were vacuumed using a vacuum pump every 24 h, followed by the daily injection of 200 mL of methane and oxygen feed in a 1:1 volumetric ratio. For all the culture enrichments, methane was used as the only carbon source in order to selectively target methane-oxidizing bacteria enrichment. The enrichment culture bottles were incubated in an orbital shaker incubator operated at a temperature of 25 °C and a mixing speed of 160 rpm. The pH was controlled at 6–7 using 10 NaOH%. The experiment was monitored by regularly measuring optical density (OD), gas consumption, and PHB accumulation after each stage of growth and PHB phase.

**Figure 1:**
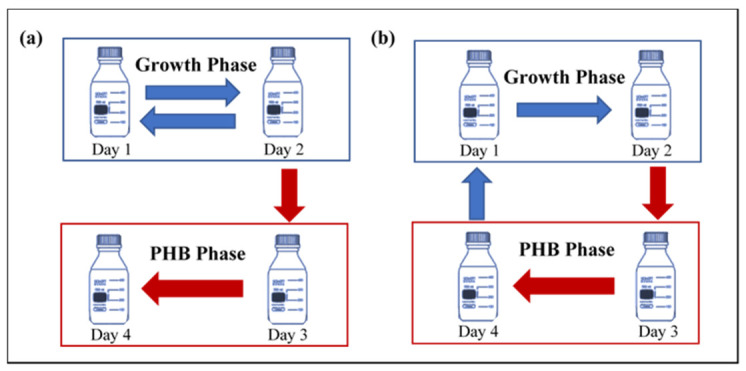
Schematic of the experimental setup. (**a**) Conventional setup (recycling of biomass after growth phase) and (**b**) new setup (recycling of biomass after PHB phase).

Consecutive cycles of fed-batch mode were implemented in this study to investigate different parameters such as growth rate, methane uptake rate, biomass yield, and microbial analysis of the prospective PHB-accumulating consortium. Four sets of culture bottles, each in triplicates, were used in this study. All sets consisted of a 96 h cycle duration comprising the growth phase and PHB accumulation phase, each of which lasted for 48 h. The growth phase media contained the nitrogen source to create a nutrient-sufficient stage aimed for bacterial enrichment. Afterward, the biomass was transferred to the PHB accumulation phase, which was devoid of any nitrogen source to create a nutrient-deficient stage in order to enable the initiation of the serine enzymatic pathway and start the selection of PHB accumulators. The first two sets were operated as control and followed a conventional setup. In the conventional setup, the cycle was started by inoculating the biomass at the beginning of the growth phase, followed by the enriched culture being split into two portions. Around 42 mL was transferred to the second stage to initiate the PHB phase, while the remaining portion was used as a seed and recycled back into the growth phase with an OD of around 0.3, as shown in Figure 1a. The other two sets were operated using the suggested new strategy, which followed the same nutrient-sufficient and nutrient-deficient alternating settings of nitrogen starvation. However, in this novel setup, the whole biomass was transferred after the growth phase into the PHB phase, and instead of recycling a portion of the biomass back after the growth phase like in the conventional setup, all the biomass was recycled after the PHB accumulation phase back to the growth phase, as shown in Figure 1b. The proposed strategy was applied to verify that the experimental design can be used as a selective pressure to preference the growth of PHB accumulators and eliminate nonproducers. Furthermore, this strategy allows different PHB producers from different strains to exist in synergy with the methane-oxidizing bacteria due the removal of nitrogen restriction. Additionally, the difference between the two sets in each setup was the type of nitrogen source, with ammonia being used in set A and nitrate in set B. 

### 2.2. Media Composition (Inoculum and Synthetic Media Composition)

A sample of recycled activated sludge (RAS) was collected from the Humber wastewater treatment plant situated in Toronto, Canada. The collected RAS was filtered using 100 μm cell filter and then centrifuged and used as a seed for culture enrichment, with 40 mL of the mineral salt media (MSM) added to each of the culture bottles. 

The MSM was prepared using the following concentrations (Bowman 2014); 1000 mg MgSO_4_.7H_2_O/L, 200 mg CaCl_2_.H_2_O/L, 272 mg KH_2_PO_4_/L, 610 mg K_2_HPO_4_/L, and 4 mg Fe-EDTA. For each bottle with 50 mL MSM media, 1 mL of trace metal solution and 250 μL of CuSO_4_ solution (corresponding to a final concentration of 1000 mg/L) were added. The trace metal solution contained the following concentrations: 10 mg ZnSO_4_.7H_2_O/L, 3 mg MnCl_2_.4H_2_O/L, 30 mg H_3_BO_3_/L, 3 mg Na2MoO_4_.2H_2_O/L, 200 mg FeSO_4_.7H_2_O/L, 2 mg NiCl_2_.6H_2_O/L, and 20 mg CoCl_2_.6H_2_O/L. Two different MSM were prepared to investigate the effect of the nitrogen source, namely ammonia salt media (ASM) for set A and nitrite salt media (NSM) for set B. In the ASM, 270 mg/L of ammonium chloride (NH_4_Cl) was added to the MSM to achieve a final concentration of 5 mM, whereas in the NSM, 1700 mg/L of sodium nitrate (NaNO_3_) was added corresponding to a final concentration of 20 mM. 

### 2.3. Analytical Techniques

#### 2.3.1. OD Measurements

A periodic measurement of the optical density (OD) for monitoring the growth of the bacterial culture in both the growth and PHB accumulation phases was performed using DR 3900 Benchtop Spectrophotometer (HACH Company, Loveland, CO, USA) at (OD_600_). A calibration curve was created to determine the correlation equation between the volatile suspended solids (*VSS*) assessed following standard protocol and the OD. The *VSS* was further used to calculate the specific growth rate (µ) and the biomass yield (Y) using Equations (1) and (2), respectively [23].

(1) $$µ=\frac{\frac{\left(VSS_{f\;}-VSS_{i}\right)}{t}}{VSS_{av.}},$$

where $VSS_{f\;}$ is the final volatile suspended solids in mg, $VSS_{i\;}$ is the initial volatile suspended solids in mg, $DCW_{av.}$ is the average of the initial and final volatile suspended solids in mg, and t is time in h.

(2) $$Y=\frac{\left(VSS_{f\;}-VSS_{i}\right)}{Methane\;Consumption},$$

#### 2.3.2. Gas Consumption

SRI 8610C gas chromatograph (SRI instrumentation, Torrance, CA, USA) equipped with a thermal conductivity detector (TCD) and molecular sieve column (Restek, Bellefonte, PA, USA) was used to measure methane, oxygen, nitrogen, and carbon dioxide concentrations. A tight glass gas syringe was used to take a gas sample from the bottle headspace, which was then injected into the gas chromatograph. The program temperature for the injector, oven, and thermal conductivity detector (TCD) were set at 60, 80, and 80 °C, respectively. Helium gas with a flow rate of 15 mL min^−1^ was used as the carrier gas. External calibration curves were established using known specific concentrations of gas mixtures in order to calculate the area under the peak and convert it into gas concentrations. The calibration was performed by vacuuming the headspace of three 250 mL serum bottles filled with 50 mL water followed by the injection of 200 mL of different gas mixtures with specific methane, oxygen, and nitrogen concentrations. Afterward, a 1 mL sample was taken from the headspace and injected in the GC instrument, which then illustrated the designated peaks used to perform calibration.

#### 2.3.3. PHB Extraction

PHB was extracted from cellular biomass and quantified using gas chromatography after each of the growth phase and the PHB accumulation phase following the protocol described with some modification as follows [24]. First, 10 mL of culture media was collected and centrifuged at 8000× *g* for 20 min at 20 °C. Then, the supernatant was decanted. Afterward, 10 mL of 5% sodium hypochlorite was added to the pellets, vortexed, and incubated for 1 h at 40 °C in a shaker incubator. Then, the hypochlorite mixture was again centrifuged, and the supernatant was decanted. Subsequently, the previous sequence of centrifuging, decanting, adding liquid, and vortexing was repeated using 10 mL of water, acetone, and ethanol. Thereupon, boiling chloroform was added to the remaining pellets and filtered through a 0.5 µm syringe filter, followed by addition of water to collect any impurities from the sample. Then, the mixture was further incubated for 2 h at 55 °C in a shaker incubator. Finally, the chloroform clear layer was transferred to another container and kept undisturbed overnight at room temperature to evaporate. Eventually, a white powdered film of the PHB polymer was formed, which was then used for further analysis.

#### 2.3.4. PHB Quantification

The PHB acquired from culture bottles was quantified using gas chromatography. First, PHB was dissolved in 2 mL chloroform and transferred to glass vial. Next, the PHB solution was kept at 100 °C for 3.5 h in a digester. Afterward, 1 mL of deionized water was added, and the mixture was vortexed for 30 s. Two phases appeared, namely an aqueous top layer and a lipophilic bottom layer. Subsequently, 1 mL was withdrawn from the bottom layer and injected into the GC for PHB quantification using SRI gas chromatography equipped with a flame ionization detector (SRI instrumentation, Torrance, CA, USA) and MXT-Wax column (Restek, Bellefonte, PA, USA). The temperature program was as follows: 1 min at 80 °C, 1 min at 100 °C, and 4 min at 180 °C. A standard curve was constructed using an external standard with known concentrations of 5, 10, and 20 mg of sodium hydroxybutyrate of 99.9% purity (Sigma Aldrich, Oakville, ON, Canada). Benzoic acid was used as an internal standard to improve accuracy of the results. The PHB concentration acquired was then divided by the total VSS in order to calculate the PHB accumulation percentage.

### 2.4. Microbial Analysis 

Two samples were obtained after the PHB phase for microbial analysis. The first sample was taken after 9 cycles of cultivation, while the second sample was taken after 16 cycles. RNA purification and amplification of the V4 region of 16S SSU rRNA were performed following the Earth Microbiome Project benchmarked protocols. The RNA purification procedure was performed with the help of the RNeasy mini-kit by QIAGEN and a clean-up step with MoBio PowerMag soil DNA isolation kit as per the manufacturer’s protocol. 

In summary, PCR amplification was carried out with a 25 µL PCR combination of deionized water, Hot Start PCR Master enzyme, 1 µL of template RNA, 0.5 mL of forward primer (10 µM), and 0.5 mL of reverse primer (10 µM). The cycle program consisted of 94 °C for 2 min, 94 °C for 30 s, 50 °C for 30 °C, 72 °C for 30 s, with a final polymerization step at 72 °C for 10 min. The amplified RNA was then quantified with Quant-KiT PicoGreen dsDNA Assay Kit (ThermoFisher, Waltham, MA, USA). Afterward, 240 ng from each sample was mixed into one tube, and the clean-up process was carried out using MoBio UltraClean PCR clean-up kit. Next, Nanodrop was used to estimate final nucleic acid concentration to guarantee that the concentration was not too little or too much and ranged from 1.8 to 2 to ensure proper sequencing. Finally, sequencing was performed using the Illumina MiSeq personal sequencer (Illumina Incorporated, San Diego, CA, USA) at the McMaster Genomics Facility, Ontario, Canada. Filtration was performed with Cutadapt and trim adapter sequences and PCR primers from the preliminary read with a quality value of at least 30 and read length of at least 100 bp. Sequence variants were then resolved from the trimmed raw reads using DADA2 to determine sequence variants. DNA sequence reads were filtered and trimmed, and taxonomy was given using the RDP classifier against the SILVA database version 1.2.8.

### 2.5. Characterization Techniques

#### 2.5.1. Sudan Black B Staining

For further confirmation of PHA accumulation, the Sudan black B staining method was performed using a counter stain to create a contrast allowing PHA observation with a light microscope with oil immersion lens 1000× magnification. First, after heat fixation of bacterial isolate, the slide was submerged with Sudan black solution (0.3% Sudan black B *w/v* in 60% ethanol). After 10 min, the slide was rinsed with distilled water, and the (0.5%) safranin counterstain was applied and left undisturbed for another 5 min. Commonly, a positive result for PHA accumulation shows dark blue scattered spots under a phase contrast microscope.

#### 2.5.2. Nucleic Magnetic Resonance (NMR)

The observed NMR spectra of a 0.7 mL sample of 16 mg of acquired PHB sample dissolved in deuterated chloroform (CDCl3) was used as a solvent at a final concentration of 10 g/L using a Bruker DXR 600 spectrometer at 24 °C with a 5 mm ^1^H-probe. The ^1^H NMR and ^13^C NMR spectra for PHB were recorded at 600 MHz and used to show correlation between proton and carbon atoms. 

## 3. Results and Discussion

A methane-oxidizing community was established after using a diluted sludge sample added to a mineral salt medium while providing methane as the sole carbon source. After four cycles of operation (start-up period), a consistent white color biomass dominated, and high methane consumption was observed. Previous studies have focused on selecting type II methanotrophs as the sole PHB producer, thus relying on high ammonium concentrations [25]. However, these studies have proven that methanotrophic bacteria on their own are incapable of achieving an accumulation percentage that can compete with other PHB producers such as *Azotobacter vinelandii* (85%) and *Pseudomonas fluorescens* (70%) [26,27]. The proposed setup aimed to increase the biomass diversity in order to allow PHB producers to coexist with the methane-oxidizing bacteria and achieve higher accumulation rates by adding a screening PHB accumulation phase that constantly recycles PHB producers back into the system.

### 3.1. Growth Rate

**Figure 2:**
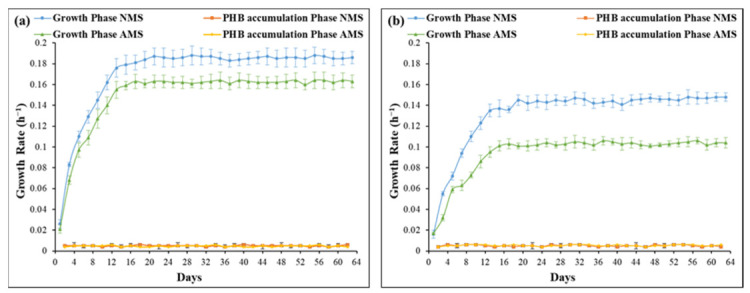
Growth rate curves for (**a**) conventional setup (recycling of biomass after growth phase) and (**b**) new setup (recycling of biomass after PHB phase).

The results of growth rates for both AMS and NMS using both the conventional and new setups in the growth phase were substantially higher than those in the PHB accumulation phase, as shown in Figure 2. This observation is in accordance with the literature and is due to the presence of all the necessary nutrients for bacterial growth, unlike in the PHB accumulation phase, where a nitrogen-deficient environment was created, which in turn hindered normal bacterial growth [28]. Moreover, it was observed that the NMS sets had higher growth rates than those of AMS in both setups. The highest specific growth rates for the NMS sets were 15% and 40% higher than those of AMS sets in the conventional and new setups, respectively. The previous results can be attributed to the fact that ammonia exhibits competitive inhibition behavior with methane substrate, which allows a smaller array of bacteria to be enriched in comparison to nitrate [20]. The methane monooxygenase (MMO) enzyme activity is reduced in ammonia presence due to the structural similarity that exists between ammonia and methane. Ammonia molecules compete with the methane molecules and attach to the enzyme’s active site, which diminishes its effectiveness in metabolizing the main substrate methane [29]. Moreover, the co-oxidation of ammonia carried out by MMO enzyme results in the production of hydroxylamine and nitrite by-products, which can cause cellular toxicity [30]. However, this toxicity occurs only in type I methanotrophs as type II methanotrophs have the ability to metabolize these by-products through the nitrification of the hydroxylamine to nitrous oxide through the action of nitric oxide reductase N_2_O from the oxidation of hydroxylamine [31]. Consequently, the relatively lower abundance of the fast-growing type I methanotrophs caused by the ammonia intoxication results in lower growth rates in the AMS sets. On the other hand, the presence of nitrate does not exert a similar inhibitory effect; in contrast, it has been reported that nitrate requires less energy because it is directly metabolized to the final product, which entails both type I and type II methanotrophs to metabolize it; hence, nitrate can be considered as a more efficient nitrogen source for bacterial growth [32]. Furthermore, the conventional setup had slightly higher specific growth rates of 0.187 and 0.163 h^−1^ for NMS and AMS, respectively, compared to 0.145 and 0.103 h^−1^ in the new setup as shown in Figure 2a,b. This is explained by the fact that in the conventional setup, bacteria are recycled after the growth phase while they are still in the exponential phase, unlike in the new setup, where biomass is recycled after they reach the stationary phase, which indicates that more time is required to re-establish a high growth rate. As shown in Figure 2a, the highest specific growth rate was reached in the NMS sets of the conventional system, which can be associated with the dominance of fast-growing type I methanotrophs in the biomass due to the absence of the competitive inhibition caused by ammonia [33]. The growth rate of mesophilic methanotrophs reported in the literature depends on the type of bacterial strain present in the culture media and has a wide range of 0.02 to 0.2 h^−1^, which is an accordance with the values obtained in this study [34,35,36].

### 3.2. Methane Uptake and Biomass Yield

**Figure 3:**
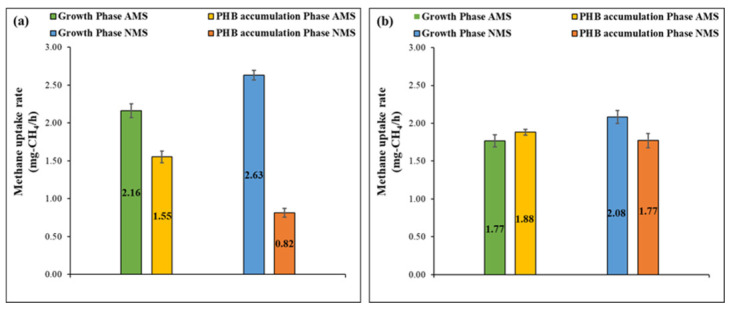
Methane uptake rate for (**a**) conventional setup (recycling of biomass after growth phase) and (**b**) new setup (recycling of biomass after PHB phase).

After the start-up period, a high methane uptake rate was recorded for all bacterial culture, which indicates the presence of methanotrophic bacteria. As shown in Figure 3, high methane uptake rates were observed for NMS sets in the growth phase for both setups, which corresponds to their higher growth rates due to the absence of the previously explained competitive inhibition behavior when nitrate is being used as the nitrogen source. As such, the methane uptake rates of the NMS sets were 2.63 ± 0.06 and 2.08 ± 0.09 mg CH_4_/h for the conventional and new setups, respectively. In comparison, the AMS sets had lower uptake rates of 2.16 ± 0.09 and 1.77 ± 0.08 mg CH_4_/h for conventional and new setups, respectively which was also in accordance with the observed growth rates. Moreover, it can be observed from the previous results that in the growth phase, the overall methane uptake rates in both sets of conventional setup were higher than those in the new setup, which may be due to the recycling strategy, which enabled bacterial biomass to consistently stay in rising growth conditions. The previous results were in the same range as that reported in the literature of 2.25–2.375 mg CH_4_/h [37].

On the other hand, in the PHB accumulation phase, the methane uptake rates were not in correlation with the observed growth rates as high methane uptake rates were measured, implying that the methane was consumed through a carbon assimilation pathway different from cellular growth. One potential pathway for methane consumption is its storage inside the bacterial cell as energy storage in the form of PHB, which usually occurs in nutrient-deficient environments as is the case in the PHB accumulation phase. Commonly, the abundance of nutrients triggers the initiation of the tricarboxylic acid (TCA) cycle for both type I and II methanotrophs either after completion of the RuMP pathway for the former or serine pathway for the latter, which in turn results in biomass growth. On the other hand, in the case of nutrient-deficient conditions, type II methanotrophs has the ability to further oxidize the glycerate resulting from the serine cycle and convert it to carbon-based intracellular polymer, i.e., PHB through the PHB cycle. Furthermore, lower methane uptake rates were observed in the PHB accumulation for the conventional setup compared to those for the new setup. The previous observation may be correlated to the difference in the selection criteria applied. In the new setup, the recycling technique, as previously mentioned, hinders the multiplication of type I methanotrophs on PHB accumulators and stimulates the growth of type II methanotrophs, which in turn consume the methane for the purpose of PHB accumulation in unfavorable conditions. On the other hand, in the conventional setup, the lack of selection pressure for PHB accumulators reflects on the reduced methane uptake in the PHB accumulation phase as the type of bacteria present in the system do not have the ability to either grow or store PHB in a nutrient-deficient environment. This is reflected in the consumption rates, with methane uptake rates of 1.88 and 1.76 mg CH_4_/h observed in the new setup compared to 1.55 and 0.82 mg CH_4_/h in the conventional setup for AMS and NMS, respectively. Moreover, it is noteworthy that the NMS set in the conventional setup resulted in considerably lower uptake rates compared to all the other sets, which is due to the absence of any PHB accumulator selection criteria due to the use of nitrate as a nitrogen source (no nitrogen-based selection) and recycling after the growth phase (no PHB-based selection).

**Figure 4:**
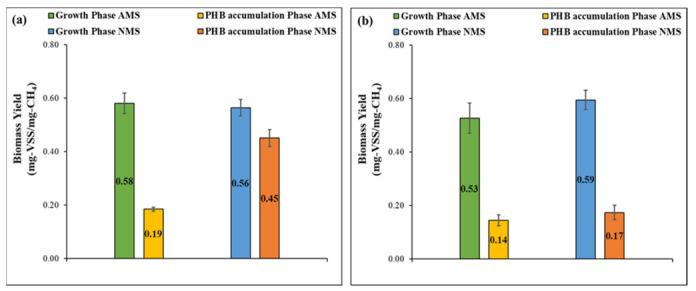
Biomass yield for (**a**) conventional setup (recycling of biomass after growth phase) and (**b**) new setup (recycling of biomass after PHB phase).

Figure 4 illustrates the relationship between the growth rate and the methane uptake rate in terms of yield. In the growth phase, the biomass yield for the four sets were comparable and ranged between 0.5 and 0.6 mg VSS/mg CH_4_, which is in accordance with the values reported in the literature for methanotrophic bacteria of around 0.4 to 0.6 mg VSS/mg CH_4_ [34,38,39]. On a similar note, the biomass yield in the growth phase was substantially higher than the PHB accumulation phase due to the low growth rate in the accumulation phase compared to the relatively high methane uptake rate. The previous observation highlights the aforementioned reduction of methane utilization in a pathway different from growth, i.e., PHB accumulation in the accumulation phase. On the other hand, high biomass yield was observed in the PHB accumulation phase except in the case of the NMS set of the conventional setup, which can be related to the corresponding low methane rate due to the absence of PHB accumulator selection where nitrate enabled bacterial growth.

### 3.3. PHB Accumulation

**Figure 5:**
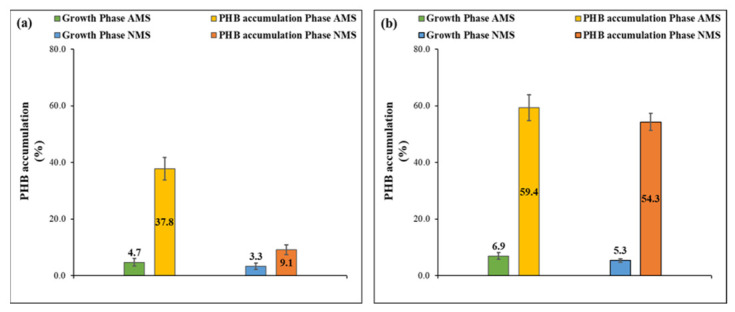
PHB accumulation percentage for (**a**) conventional setup (recycling of biomass after growth phase) and (**b**) new setup (recycling of biomass after PHB phase).

In the first few cycles of the experiment, almost no polymer was observed; however, as the experiment progressed, a gradual increase in PHB accumulation measurements was noticed in all sets. After the fifth cycle of operation, the extracted polymer amount stabilized and was in a constant range within subsequent cycles. As shown in Figure 5, very low PHB accumulation percentages were recorded in the growth phase in the AMS and NMS sets of both setups, ranging between 3.3% and 6.9%. The previous observation is due to the abundance of nitrogen in the growth phase, which contradicts the fact that PHB storage is initiated as a result of the stress conditions induced by nitrogen deficiency applied in the subsequent PHB phase. The PHB cycle is usually intercorrelated with the serine cycle in type II methanotrophs. PHB accumulation is normally initiated with a spontaneous reaction occurring by conversion of formaldehyde through a designated pathway into 5,10-methylene-tetrahydrofolate (MTF). which combines with one carbon dioxide molecule to produce the amino acid serine [40,41]. Through a set of orchestrated phosphorylation reactions, serine is further converted into glycerate, phosphoenolpyruvate, malate, malyl-CoA, and finally glyoxylate [16]. It is worth mentioning that the glyoxylate regeneration cycle (GRC) links the serine cycle to the TCA and PHB cycles by constantly converting and regenerating the acetyl coenzyme A to glyoxylate and constantly feeding it back into the serine cycle [40,42]. The glyoxylate and malyl-CoA are incorporated in the TCA by conversion to isocitrate of the former and acetyl-CoA of the latter, which are considered terminal products of the serine cycle. In the case of the absence of nutrients essential for TCA cycle completion, the PHB cycle is triggered to store an alternative supply of energy that acts as a reducing potential for carbon assimilation when nitrogen is depleted [15]. Simultaneously, an important carbon assimilatory pathway is integrated with the PHB cycle ethyl malonyl-CoA (EMC) pathway, which encompasses several CoA derivatives to deliver a 3-hydroxybutyryl-CoA molecule. Coenzyme A is released through a stepwise catalytic pathway that takes place in order to form the hydroxybutyrate monomer with the help of a set of enzymes expressed by some specifically PHB-related genetic codes called phaA, phaB, and phaC that express the enzymes β-ketothiolase, acetoacetyl-coA reductase, and PHB synthase, respectively [43]. Thus, the 3-hydroxy-butyryl (HB) monomer is accordingly added to the polymeric chain and stored intracellularly in a granular form. On the other hand, in nutrient-sufficient conditions after the serine cycle, instead of undergoing the PHB pathway, the acetyl Co-A produced combines with another acetyl Co-A molecule to produce citrate. This marks the start of the tricarboxylic acid cycle to produce energy for biomass synthesis through the electron transport chain, thus explaining the low PHB accumulation in the growth phase and high accumulation in the PHB phase.

As illustrated in Figure 5, the highest PHB accumulation percentages were attained in the AMS and NMS sets of the new setup and were equal to 59.4 ± 4.5% and 54.3 ± 3%, respectively, compared to 37.8 ± 4% and 9.1 ± 1.8% in the AMS and NMS sets of the conventional setup, respectively. This demonstrates the success of the new setup in exceeding the reported threshold accumulation of 40–51% of the media-based selection [21,22]. Moreover, the 600% increase in PHB accumulation in the NMS sets in the new setup compared to the conventional setup proves the tested hypothesis of the new methodology that the PHA-accumulating methanotrophic consortium can grow without the restriction of a specific nitrogen source, i.e., ammonia. The previous conclusion is also demonstrated by the comparable PHB accumulation results for ammonia and nitrate as nitrogen source in the new setup. 

### 3.4. Microbial Analysis

**Figure 6:**
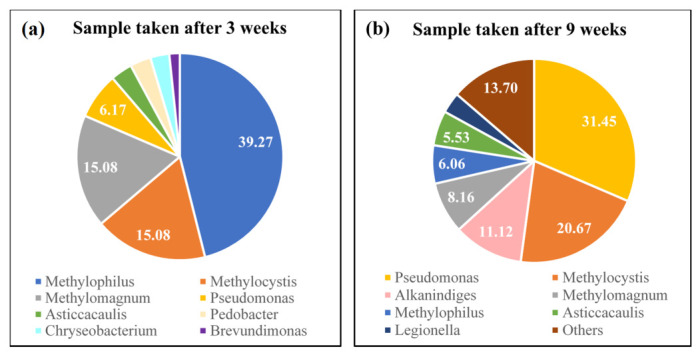
Microbial analysis at the genus level for samples taken from the NMS-enriched biomass of the new setup. (**a**) Sample taken after 3 weeks of enrichment and (**b**) sample taken after 9 weeks of enrichment.

Two samples taken after 3 and 9 weeks of experimentation from the NMS set of the new setup were sent for sequencing in order to identify the structure of the microbial community. The microbial analysis of the first sample (taken after 3 weeks) showed a dominance of non-PHB accumulating type I methanotrophic bacteria represented by the *Methylophilus* and *Methylomagnum* genus, which accounted for 39.27% and 15.08% of the total microbial community, as illustrated in Figure 6a. In contrast, type II methanotrophs, which are known for their PHB accumulation ability, constituted only 15.08% of the total community and was represented by the *Methylocystis* genus. On the other hand, the sequencing of the sample taken after 9 weeks of experimentation (equivalent to 16 operational cycles) showed a shift in the microbial community from a dominance of Gammaproteobacteria type I methanotrophs to a dominance of *Pseudomonas* genus, which accounted for 31.45% of the total community, as illustrated in Figure 6b. Pseudomonas bacteria are able to feed on a variety of carbon substrates and are well known for their PHB accumulation capability as they can accumulate more than 80% of their cell dry weight [44,45]. Methanotrophic bacteria are known to produce a variety of different by-products, many of which can act as substrate for the Pseudomonas bacteria, such as organic acids, fatty acids, and exopolysaccharides. These compounds can take part in metabolic pathways, such as glycolysis and glyoxylate shunt, citric acid cycle, or B-oxidation [46]. Furthermore, type II methanotrophs also witnessed a 37% increase, reaching around 21%, and were represented by the *Methylocystis* genus. On the other hand, the increase in PHB producers was accompanied by a decrease in non-PHB accumulating type I methanotrophs from 55% to only 6% of the total microbial community. The substantial increase in *Pseudomonas* and type II methanotrophs accompanied by the decrease in type I methanotrophs can in turn explain the high PHB accumulation percentage acquired in the new setup. This shift also confirms that the newly proposed selection-based design successfully screened for PHB accumulators by creating an environment where type II methanotrophs could coexist with other PHB accumulators that have the ability to uptake metabolic by-products of methanotrophic bacteria and use them as substrate in a methane-rich environment.

### 3.5. PHB Characterization

#### 3.5.1. Sudan Black and Extracted Powder

Sudan Black B dye is a lipophilic dye that attaches to the ester bond in the polymeric chain of the PHB granule and is used for the preliminary detection of PHA lipid inclusions [47]. The absorption of this lipophilic dye is reflected by the observation of scattered dark blue spots, which confirms the presence of PHA granules within the microbial cells, as shown Figure S2. 

**Figure 7:**
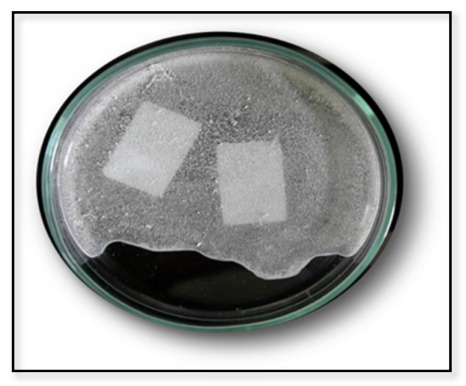
The extracted PHB polymer powder with the sodium hypochlorite method.

PHA extraction was carried out with sodium hypochlorite protocol, which is crucial for further characterization of the biopolymer with other analytical techniques [25]. High-purity extraction was achieved and confirmed by the NMR spectra by adding multiple steps, washing, and polymer filtration. The extracted polymer was in the form of a white powder, as shown in Figure 7.

#### 3.5.2. Nuclear Magnetic Resonance (NMR)

**Figure 8:**
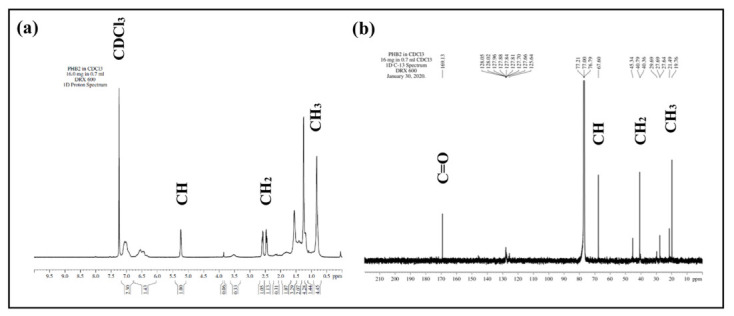
NMR for extracted PHB polymer. (**a**) Proton NMR and (**b**) carbon NMR.

The proton and carbon NMR indicated the presence of different functional groups at varying chemical shifts, as shown in Figure 8 (C=O, CH, CH_2_, and CH_3_). The NMR spectra for the PHB polymer was in accordance with the PHB spectra recorded by Jiang et al. (2008), which confirms the structural formula for the acquired polymer [28]. 

The peak number 1 was observed at 1.2 and 19.76 ppm for ^1^H NMR and ^13^C NMR, respectively, and was assigned to terminal methyl proton CH_3_. The multiple peak number 2 at the range of 2.4–2.6 ppm and 40.79 ppm was assigned to methylene protons CH_2_ adjacent to the CH carbon in the sidechain. Peak number 3 at 5.2 and 67.6 ppm was assigned to CH=CO proton. Peak number 4 at 169.13 ppm in ^13^C NMR was assigned to the carbonyl group C=O, as shown in Table 1. 

## 4. Conclusions

A comprehensive comparison was made between two experimental setups for favoring the growth of PHB accumulators, namely the conventional ammonia-dependent setup and the newly proposed ammonia-independent setup, which relies solely on the recycling technique. In this experiment, nitrogen limitation was the main precursor for the initiation of the PHB accumulation process, which was achieved through two cultivation stages, i.e., the growth phase (nitrogen rich) and the PHB accumulation phase (nitrogen limited). Furthermore, two nitrogen sources were employed as the main objective was to attain high PHB accumulation percentage that is analogous to that of ammonia’s, reinforcing the concept that ammonia as a nitrogen source is not essential for the selection of type II PHB accumulators. The success of the newly proposed setup was confirmed by achieving the highest PHB accumulation percentage recorded for a mixed culture community in both AMS and NMS media of 59.4% and 54.3%, respectively, compared to 37.8% and 9.1% for the conventional setup. The relatively small difference in PHB accumulation achieved in the new setup when using ammonia and nitrate as nitrogen sources demonstrates the success of the new recycle technique for a mixed culture community in achieving high PHB accumulation independently of the nitrogen source used. These results could not be achieved in the conventional setup, where the use of ammonia as the nitrogen source resulted in more than 4 times higher PHB accumulation than with nitrate. Furthermore, the microbial analysis showed a significant increase in the ratio of type II methanotrophic bacteria after 9 weeks of experimentation, unlike the samples taken after 3 weeks of experimentation, which was dominated by type I methanotrophs. Moreover, a microbial shift toward PHB accumulators was established through the enrichment of other PHB producers that coexisted with type II methanotrophs. This interesting finding opens the door for the enrichment of a diversity of microbial groups that can support the presence of methanotrophic bacteria either by removing toxic by-products, thereby enhancing their metabolic activities, or by accumulating PHB within their own cells, which can help supersede the threshold accumulation recorded for methanotrophic bacteria in the literature. 

## Figures and Tables

**Table 1:** The chemical groups and their chemical shift signals for ^13^C NMR and ^1^H NMR spectra.

Functional Groups	^13^C NMR (ppm)	^1^H NMR (ppm)
CH_3_	19.76	1.2
CH_2_	40.79	2.4–2.6
CH	67.60	5.2
C=O	169.13	-

## Data Availability

The data presented in this study are available in this manuscript.

## Supplementary Materials

The following are available online at https://www.mdpi.com/article/10.3390/polym13101579/s1, Figure S1: An illustration of the competitive inhibition between ammonia and methane, Figure S2: PHB granules observed under confocal microscope using Sudan Black B dye.
