# Supplementary material for: Screening for Methane Utilizing Mixed Communities with High Polyhydroxybutyrate (PHB) Production Capacity Using Different Design Approaches
Source: Polymers (Basel). 2021 May 14;13(10):1579. doi: 10.3390/polym13101579 (PMC8157138; doi:10.3390/polym13101579)
Supplement: Supplementary file 1 [file polymers-13-01579-s001.zip › polymers-1142946-supplementary.pdf]

polymers

MDPI

Supplementary Information

Screening for Methane Utilizing Mixed Communities with High Polyhydroxybutyrate (PHB) Production Capacity Using Different Design Approaches

Rana Salem [1], Moomen Soliman [2], Ahmed Fergala [3], Gerald F. Audette [4] and Ahmed ElDyasti [5,*]

[1] Civil Engineering Department, York University, 4700 Keele street, Toronto, M3J 1P3, ON, Canada; rsalem@yorku.ca
[2] Civil Engineering Department, York University, 4700 Keele street, Toronto, M3J 1P3, ON, Canada; ilasimbf@yorku.ca
[3] Department of Biotechnology, Delft University of Technology,Mekelweg 5, 2628 CD Delft, Netherlands; a.m.a.a.fergala@tudelft.nl
[4] Chemistry Department, York University, 4700 Keele street, Toronto, M3J 1P3, ON, Canada; audette@yorku.ca
[5] Civil Engineering Department, York University, 4700 Keele street, Toronto, M3J 1P3, ON, Canada; ahmed.eldyasti@lassonde.yorku.ca
* Correspondence: ahmed.eldyasti@lassonde.yorku.ca

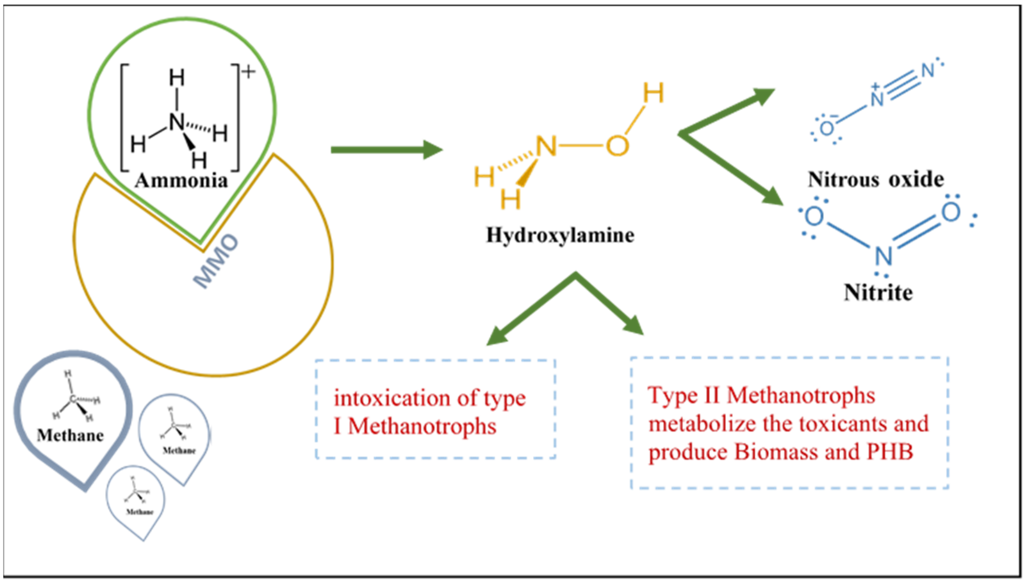

Figure S1: An illustration of the competitive inhibition between ammonia and methane

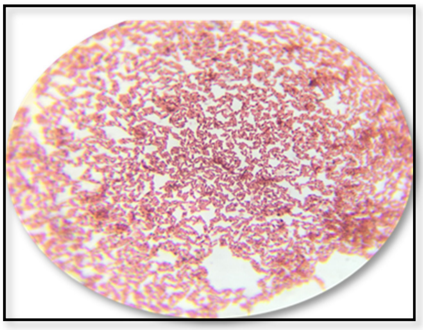

Figure S2: PHB granules observed under confocal microscope using Sudan Black B dye.
